# Functional and Histological Analysis of Stem Cell and Amniotic Membrane Implantation After Acute Myocardial Infarction with Left Ventricular Dysfunction: Experimental Study

**DOI:** 10.3390/ijms27083397

**Published:** 2026-04-10

**Authors:** Isabella Cristina Mendes Rossa, Marcos Antônio Denk, Luize Kremer Gamba, Anna Clara Faidiga Silva, Julia Letícia de Bortolo, Igor Ramos Lima, Paulo Cesar Lock Silveira, Eltyeb Abdelwahid, Márcia Olandoski, Júlio Cesar Bassan, Lucia de Noronha, Júlio Cesar Francisco, Luiz César Guarita-Souza

**Affiliations:** 1 Graduate Program in Health Sciences, School of Medicine, Pontifícia Universidade Católica do Paraná (PUCPR), Curitiba 80215-901, Brazil; slidesdenk@gmail.com (M.A.D.); luizekremer@hotmail.com (L.K.G.); annafaidiga@hotmail.com (A.C.F.S.); jldbortolo@gmail.com (J.L.d.B.); marcia.olandoski@pucpr.br (M.O.); lucia.noronha@pucpr.br (L.d.N.); julio.francisco1@pucpr.br (J.C.F.); cesar.souza@pucpr.br (L.C.G.-S.); 2 Laboratory of Experimental Physiopathology, Program of Postgraduate in Science of Health, Universidade do Extremo Sul Catarinense, Criciúma 88806-000, Brazil; igor.mamp@unesc.net (I.R.L.); psilveira@unesc.net (P.C.L.S.); 3 Department of Medicine, University of Illinois Chicago, Chicago, IL 60612, USA; elteba@uic.edu; 4 Postgraduate Program in Physical Education, Universidade Tecnológica Federal do Paraná, Curitiba 81310900, Brazil; jcbassan@gmail.com; 5 Postgraduate Program in Biomedical Engineering (PPGEB-CT), Universidade Tecnológica Federal do Paraná, Curitiba 81310900, Brazil

**Keywords:** acute myocardial infarction, heart failure, mononuclear stem cells, human amniotic membrane, tissue regeneration

## Abstract

Acute myocardial infarction (AMI) results from a lack of oxygen supply to the myocardium, leading to the loss of cardiomyocytes and their replacement with fibrotic scar tissue. This process is closely associated with the development of heart failure. Regenerative medicine has emerged as a promising strategy to enhance treatment outcomes in severe cases of heart failure. This study aimed to evaluate myocardial regeneration after AMI using a biomaterial composed of mononuclear stem cells and human amniotic membrane. A total of 120 Wistar rats were subjected to experimentally induced AMI. On the 7th day post-infarction, rats with an ejection fraction of <50% on echocardiography were randomized into four groups: (1) control; (2) stem cells; (3) amniotic membrane; and (4) amniotic membrane combined with stem cells. On the 30th day, the surviving animals underwent a second echocardiographic evaluation and were subsequently euthanized. The group treated with the combination of amniotic membrane and stem cells showed reduced systolic and diastolic ventricular volumes. Histological analysis revealed that these animals exhibited less fibrosis and a lower percentage of type I collagen. Based on the results of the study, it was concluded that the combination of human amniotic membrane and mononuclear stem cells decreased ventricular volumes and myocardial fibrosis, suggesting more favorable ventricular remodeling in this experimental model.

## 1. Introduction

Acute myocardial infarction (AMI) is recognized as the leading single cause of death from cardiovascular disease worldwide. Myocardial necrosis involves the death of cardiomyocytes and their subsequent replacement with fibrotic scar tissue, a process known as ventricular remodeling [1,2].

Following acute myocardial infarction, extensive cardiomyocyte loss triggers a reparative process characterized by inflammatory activation and progressive replacement of necrotic tissue with fibrotic scar formation. Although fibrosis provides essential mechanical stability to prevent ventricular wall rupture, the resulting extracellular matrix deposition increases myocardial stiffness, impairs contractility, and disrupts electrical conduction. Over time, persistent or excessive fibrotic remodeling contributes to adverse ventricular dilation and progression to heart failure, highlighting fibrosis as both a structural safeguard and a key determinant of long-term functional deterioration [3].

Therefore, strategies capable of modulating post-infarction fibrosis while preserving structural integrity represent a promising therapeutic avenue in cardiac regenerative medicine.

Taken together, these findings are in line with emerging regenerative strategies that seek to simultaneously address structural damage and electrophysiological instability after myocardial infarction. Recent work on ion cocktail therapy, for example, has demonstrated that coordinated modulation of ionic homeostasis can improve both structural and electrical remodeling in infarcted hearts, supporting the rationale for multifaceted interventions in this setting [4].

Chronic ventricular remodeling represents a maladaptive response and contributes significantly to ventricular dysfunction [4,5]. This process alters the size and shape of the heart, leads to regions of myocardial dyskinesia or akinesia, and causes progressive deterioration of ventricular function, hallmarks that contribute to the development of heart failure (HF) [4].

Heart failure is a complex clinical syndrome that is difficult to manage. It significantly impairs quality of life and is associated with reduced life expectancy. Common symptoms include progressive dyspnea, orthopnea, paroxysmal nocturnal dyspnea, peripheral edema, and exercise intolerance [5,6].

Despite advances in medical management, HF remains a condition with high morbidity and mortality, particularly in advanced stages where treatment options are limited to mechanical circulatory support and heart transplantation [7]. The limited effectiveness of existing therapies underscores the urgent need for novel approaches to mitigate the consequences of this disease.

One such approach involves the use of the human amniotic membrane (HAM) in infarcted hearts. HAM has gained attention due to its potential regenerative effects, not only through cellular transdifferentiation but also because of its anti-inflammatory, bacteriostatic, and antimicrobial properties [8].

Research suggests that HAM benefits are largely mediated by the release of immunomodulatory and anti-inflammatory cytokines, such as interleukins 6 and 10 [9]. Additionally, tissue repair may be enhanced by the secretion of angiogenic factors, including vascular endothelial growth factor (VEGF), angiogenin, and platelet-derived growth factor (PDGF), which play key roles in tissue healing [10]. However, larger translational studies are required to validate the cost-effectiveness and clinical applicability of this technique [8].

Furthermore, mononuclear stem cells have emerged as a potential therapeutic tool for ischemic myocardial injury. In 2001, Orlic et al. proposed that human bone marrow cells could regenerate myocardial tissue following AMI [11]. Since then, numerous studies have explored the use of various cell types, molecules, and genes to modulate local and systemic inflammation, reduce apoptosis, promote angiogenesis, and generate new contractile cardiomyocytes, while preserving the functional and structural integrity of the extracellular matrix [12].

In this context, biomaterial scaffolds combined with stem cells may offer a synergistic strategy to enhance myocardial repair by providing structural support and modulating the inflammatory and fibrotic microenvironment. Therefore, the aim of this study was to evaluate myocardial regeneration after AMI using a biomaterial composed of mononuclear stem cells associated with human amniotic membrane.

## 2. Results

### 2.1. Functional Analysis

The analysis presented below was based on data from 43 rats with EF < 50%, randomized to one of 4 groups according to treatment: Control group (*n* = 12), SC group (*n* = 9), HAM group (*n* = 10) and HAM + SC group (*n* = 12). All the animals were assessed at two points in time: day 7 and day 30.

Table 1 shows the homogeneity of the data when comparing the groups on day 7 of the study, as no significant differences were found for the variables EF (%; *p* = 0.349), SV (mL; *p* = 0.349) and DV (mL; *p* = 0.533). For the results obtained on the 30th day of the study, the analysis of EF (%) revealed no statistical difference when comparing the groups (*p* = 0.072). Statistical differences were found for SV (mL) and DV (mL), with *p* = 0.023 and *p* = 0.05, respectively.

In the analysis between day 7 and day 30 of the study, there was a reduction in mean EF values (%) in the Control [(37.3 ± 5.6 ➝ 36.5 ± 5.7); *p* = 0.656] and SC [(38.9 ± 6.5 ➝ 33.3 ± 7.1); *p* = 0.096] and an increase in the HAM [(35.1 ± 5.9 ➝ 37.8 ± 9.3; *p* = 0.126] and HAM + SC [(37.9 ± 6 ➝ 42.2 ± 9); *p* = 0.258] groups, but there was no statistical significance for any of the values described.

With regard to the variable Systolic Volume (mL), there was an increase in the mean values in the Control [(0.159 ± 0.046 ➝ 0.194 ± 0.07); *p* = 0.101] and HAM [(0.143 ± 0.067 ➝ 0.194 ± 0.071; *p* = 0.067), as well as a reduction in the SC [(0.191 ± 0.066 ➝ 0.156 ± 0.075); *p* = 0.348] and HAM + SC [(0.177 ± 0.067 ➝ 0.118 ± 0.068); *p* = 0.034] groups, with significant values found only for the HAM + SC group (*p* = 0.034).

For the DV (mL) variable, there was an increase in the mean values between day 7 and 30 in the Control [(0.257 ± 0.065 ➝ 0.301 ± 0.079); *p* = 0.086] and HAM [(0.257 ± 0.142 ➝ 0.313 ± 0.077); *p* = 0.228], as well as a reduction in the SC [(0.315 ± 0.098 ➝ 0.229 ± 0.105); *p* = 0.161] and HAM + SC [(0.287 ± 0.096 ➝ 0.197 ± 0.084); *p* = 0.013] groups, with significant values found only for the HAM + SC group (*p* = 0.013).

Analysis of the SV (mL) and DV (mL) variables on the 30th day of the study found significant differences between the study groups, with a *p*-value of 0.023 and 0.005, respectively. The groups were therefore compared two by two for these variables, as shown in Table 2.

According to the results in Table 2, significant differences were found when comparing the Control x (HAM + SC) groups for the DV (mL; *p*= 0.031) and SV (mL; *p* = 0.05) variables. In addition, there was also statistical significance when comparing the HAM x (HAM + SC) groups for the DV (mL; *p* = 0.019) variables.

### 2.2. Histological Analysis

**Figure 1:**
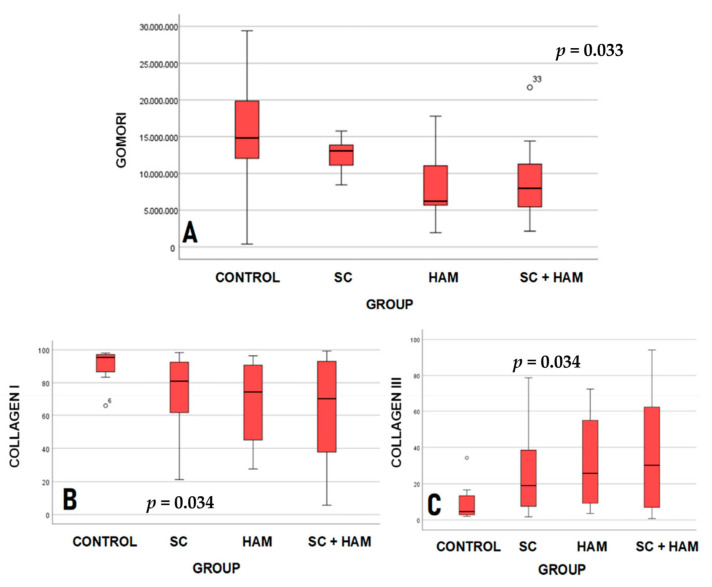
Box-plot representing the analysis of the variables Gomori Trichrome (**A**), Collagen type I (**B**) and III (**C**) in Control, SC, HAM and SC + HAM groups. Legend: HAM: Human Amniotic Membrane; SC: Stem Cells.

Table 3 and Figure 1 show the results of the comparison between the study groups for the variables Gomori trichrome (µm^2^), type I collagen (%) and type III collagen (%).

According to Table 3, the values obtained from the Gomori Trichrome analysis indicate that the mean infarct areas for the Control (15,585,072 µm^2^ ± 7,997,958) and SC (12,810,354 µm^2^ ± 2,452,308) groups were higher than the means for the HAM (8,890,165 µm^2^ ± 5,613,027) and HAM + SC (8,961,009 µm^2^ ± 5,283,955) groups, and statistical significance was found for this analysis (*p* = 0.033).

With regard to the results obtained from the Sirius-red analysis, it can be inferred that the mean percentages of type I collagen for the Control (91% ± 9.9) and SC (74.6% ± 23.9) groups were higher than the values for the HAM (66.7% ± 25.7) and HAM + SC (63.1% ± 32.3) groups. A different pattern was found with the type III collagen averages (%), as the values found for the HAM (33.3% ± 25.7) and HAM + SC (36.9% ± 32.3) groups were higher compared to the Control and SC groups. There was statistical significance for the values obtained for both the percentages of type I collagen (*p* = 0.034) and type III collagen (*p* = 0.034).

For the groups analyzed, significant differences were found in the analysis of the variables Gomori trichrome (µm^2^; *p* = 0.033), and type I collagen (%; *p* = 0.034) and type III collagen (%; *p* = 0.034). The groups were then compared two by two. Table 4 shows the *p*-values for these comparisons.

The results in Table 4 indicate that significant differences were found when comparing the Control and (HAM + SC) groups for the variables Gomori trichrome (µm^2^; *p*= 0.05), type I collagen (%, *p* = 0.05) and type III collagen (%, *p* = 0.05).

**Figure 2:**
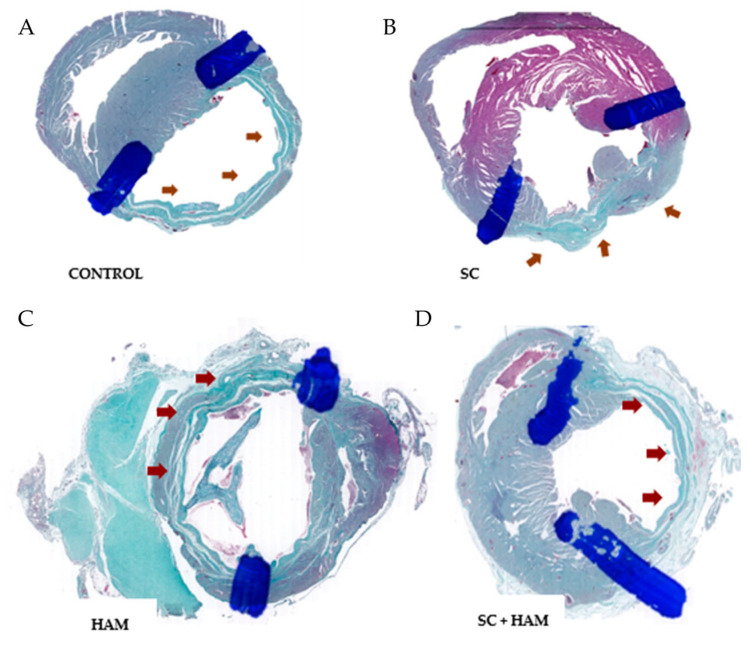
Gomori trichrome staining of myocardial tissue in Control, SC, HAM, and SC + HAM groups. (**A**) Control: extensive fibrosis (blue) in the infarct region. (**B**) SC: moderate fibrosis reduction. (**C**) HAM: further reduction in collagen deposition. (**D**) SC + HAM: minimal fibrosis and better preserved myocardium. Magnification 400×; scale bar = 25 µm. Blue: collagen/fibrosis; red: myocardium.

In Gomori’s trichrome staining, the infarct area can be identified by the light blue marking, as shown in Figure 2. 

**Figure 3:**
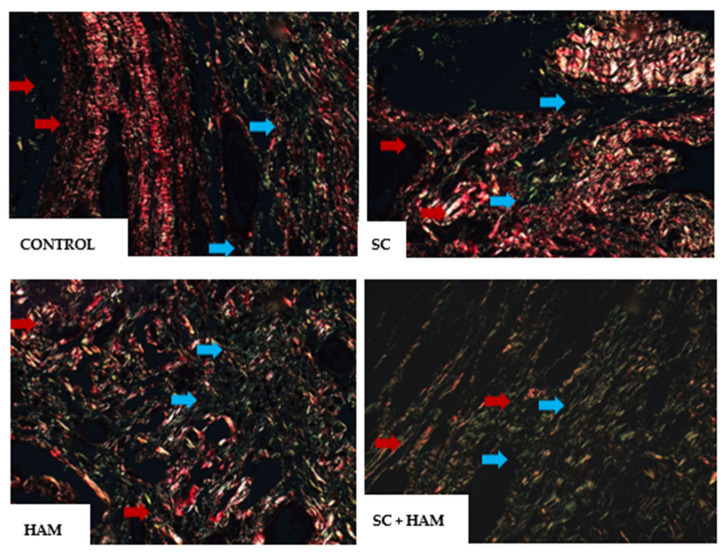
Visualization of type I collagen (in red—pointed by the red arrow) and type III collagen (in green—blue arrow) in optical microscopy of myocardial tissue slide using Sirius-red staining, in Control, SC, HAM and SC + HAM groups, respectively. The images were obtained from the infarction zone. Magnification 400×; white scale bar = 25 µm. Legend: HAM: Human Amniotic Membrane; SC: Stem Cells.

In Sirius-red staining, type I collagen can be identified by the red color and type III collagen by the green color, as shown in Figure 3:

### 2.3. Immunohistochemical Analysis

**Figure 4:**
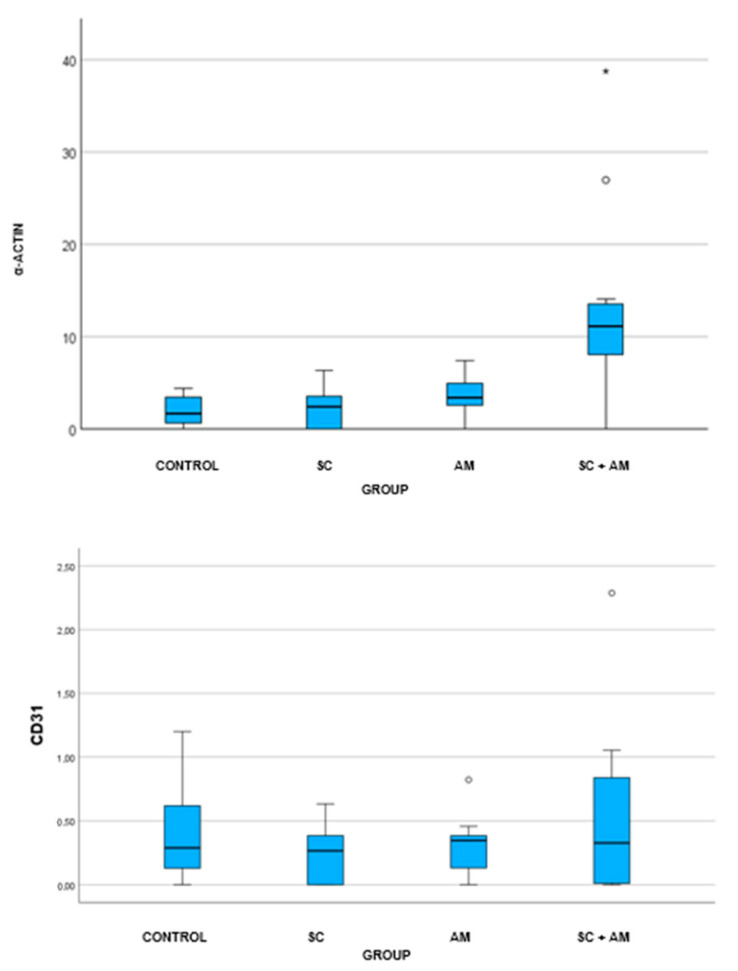

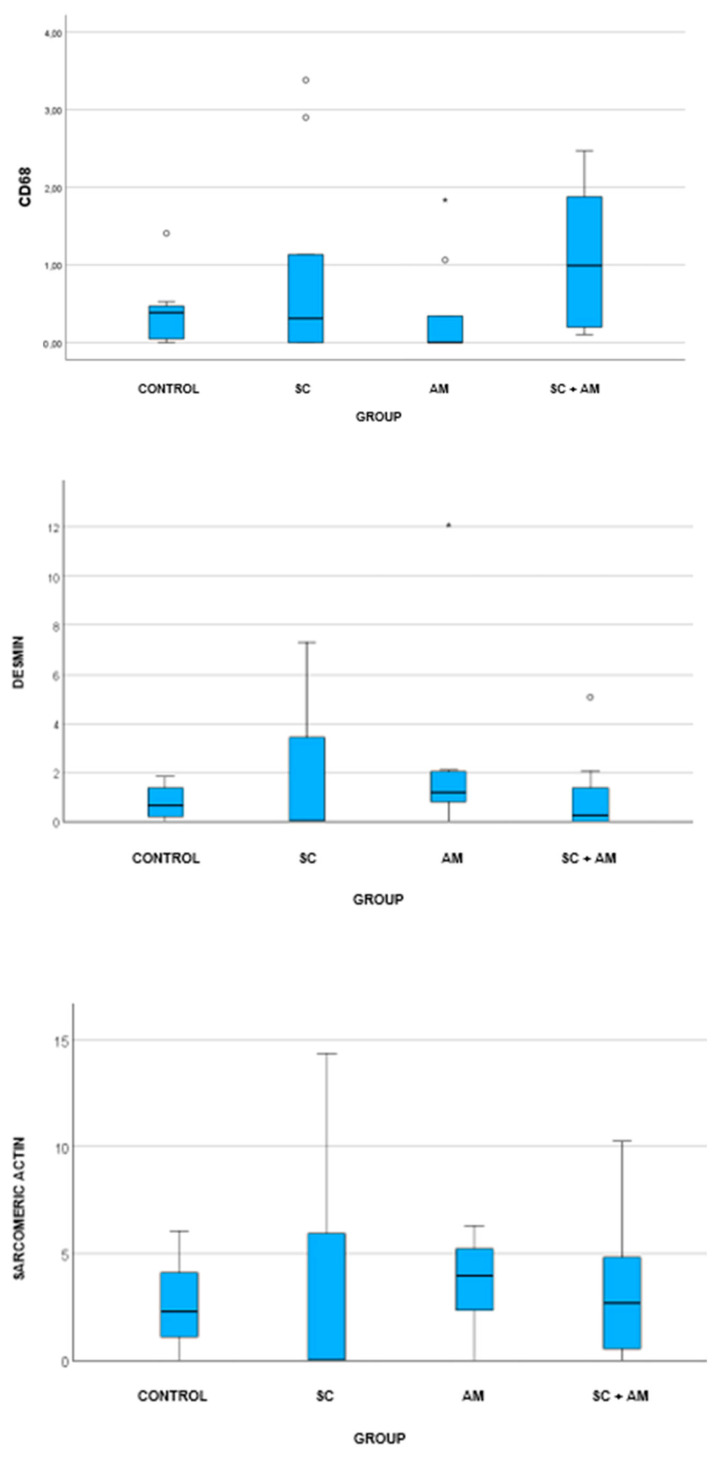
Box-plot representing the analysis of the variables α-actin, CD31, CD68, desmin and sarcomeric actin between the study groups, in Control, SC, HAM and SC + HAM groups. Legend: HAM: amniotic membrane; SC: Stem Cells; *: outlier.

The markers used for immunohistochemical analysis of the myocardial slides were: α-actin, CD31, CD68, desmin and sarcomeric actin. Table 5 and Figure 4 show the results obtained from the analysis of these markers.

#### 2.3.1. α-ACTIN

Table 5 and Figure 2 show the results obtained from the analysis of the α-actin (%) variable. The mean values obtained for the (HAM + SC) group (13.32% ± 10.21) were higher than those for the Control (1.97% ± 1.61), SC (2.38% ± 2.2) and HAM (3.49% ± 2.19) groups, and statistical significance was found for the results (*p* < 0.001).

Significant differences were found between the groups for the α-actin variable (%) (*p* < 0.001). The study groups were therefore compared two by two. Table 6 shows that the comparison:

Table 6 shows that there was a significant difference when comparing the α-actin variable (%) for the following groups: Control x (HAM + SC) [*p* = 0.001], SC x (HAM + SC) [*p* = 0.003] and HAM x (HAM + SC) [*p* = 0.066].

#### 2.3.2. CD31

Analysis of the results obtained for the CD31 variable (%) in Table 5 and Figure 2 shows that the median values obtained for the Control (0.44 ± 0.43) and HAM + SC (0.55 ± 0.67) groups were higher than those found for the HAM (0.32 ± 0.25) and SC (0.24 ± 0.23) groups, where there was no statistical significance to the results obtained (*p* = 0.743).

#### 2.3.3. CD68

Analysis of CD68 indicated that the mean values obtained for the HAM + SC group (1.09 ± 0.89) were higher than those found for the Control (0.37 ± 0.40), SC (0.94 ± 1.30) and HAM (0.36 ± 0.66) groups, where there was no statistical significance for the values found (*p* = 0.061), as shown in Table 5 and Figure 2.

#### 2.3.4. Desmin

Table 5 and Figure 2 show the values obtained from the analysis of the desmin marker (%), which indicate that the average obtained for the HAM group (2.37 ± 3.71) was higher than that obtained in the HAM + SC (0.91 ± 1.51), Control (0.80 ± 0.71) and SC (1.77 ± 2.83) groups, where there was no statistical significance for the values found (*p* = 0.264).

#### 2.3.5. Sarcomeric Actin

The analysis of sarcomeric actin indicated that the mean values obtained for the SC group (4.02 ± 5.43) were higher than those of the HAM (3.55 ± 2.30), Control (2.58 ± 2.02) and HAM + SC (3.28 ± 3.15) groups, in which there was no statistical significance for the values found (*p* = 0.755), as shown in Table 5 and Figure 2.

**Figure 5:**
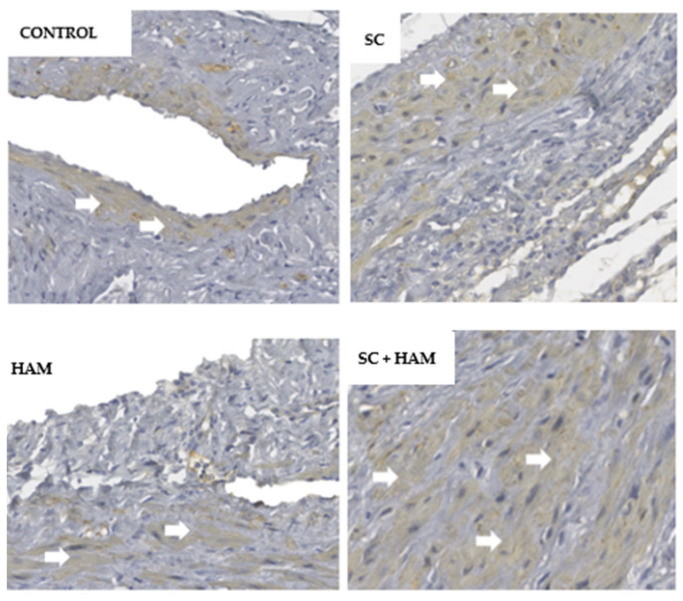
Visualization of the α-actin marker (white arrow) on a myocardial tissue slide under light microscopy, in Control, SC, HAM and SC + HAM groups, respectively. The images were obtained from the infarction zone. Magnification: 200×. Scale bar: 50 μm. Legend: HAM: Human Amniotic Membrane; SC: Stem Cells.

**Figure 6:**
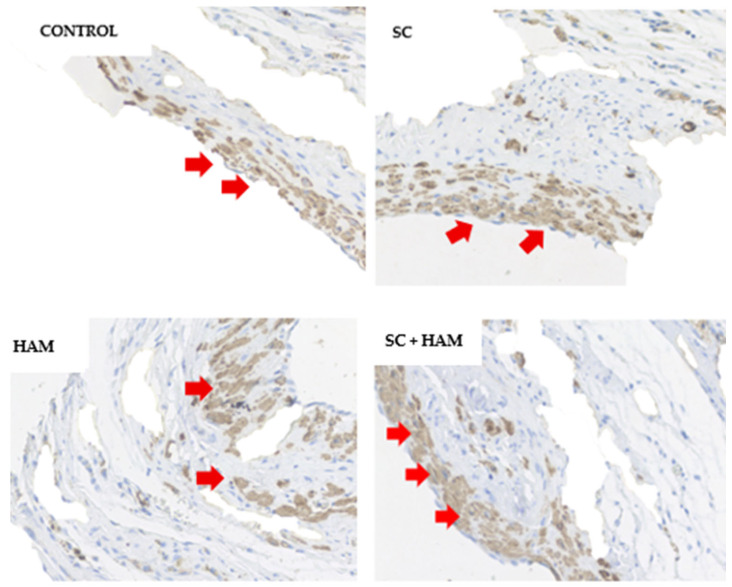
Visualization of the sarcomeric actin marker (red arrow) on a myocardial tissue slide under light microscopy, in Control, SC, HAM and SC + HAM groups, respectively. The images were obtained from the infarction zone. Magnification: 400×. White scale bar: 25 μm. Legend: HAM: Human Amniotic Membrane; SC: Stem Cells.

Regions with expression compatible with the α-actin marker can be identified by brown coloring, as observed in Figure 5 and with the sarcomeric actin marker in Figure 6.

## 3. Discussion

This study aimed to carry out a functional and histological analysis of the implantation of mononuclear stem cells and human amniotic membrane after acute myocardial infarction with left ventricular dysfunction.

With regard to the functional analysis, the results obtained on the 30th day of the study for the EF variable (%) showed no statistical significance when comparing the groups (*p* = 0.072). Statistical differences were found for the SV (mL) and DV (mL) variables, with *p* = 0.023 and *p* = 0.05, respectively (Table 1).

In the analysis between day 7 and day 30 of the study, there was a reduction in mean EF values (%) in the Control (*p* = 0.656) and SC (*p* = 0.096) groups and an increase in the AM (*p* = 0.126) and AM + SC (*p* = 0.258) groups.

With regard to the variable Systolic Volume (mL), there was an increase in the mean values in the Control (*p* = 0.101) and HAM (*p* = 0.067), as well as a reduction in the SC (*p* = 0.348) and HAM + SC (*p* = 0.034) groups, with significant values found only for the HAM + SC group (*p* = 0.034).

The analysis of the SV variable (mL) on day 30 found significant differences between the study groups, with *p*-values of 0.023. The groups were then compared two by two for this variable. Significant differences were found when comparing the Control and (HAM + SC) groups, with a *p*-value of 0.005.

For the DV (mL) variable, there was an increase in the mean values between day 7 and 30 in the Control (*p* = 0.086) and HAM (*p* = 0.228), as well as a reduction in the SC (*p* = 0.161) and HAM + SC (*p* = 0.013) groups, with significant values found only for the HAM + SC group (*p* = 0.013). In the analysis of the DV (mL) variable on day 30, significant differences were found between the study groups, with a *p*-value of 0.005. The groups were therefore compared two by two for these variables. Significant differences were found when comparing the Control x (HAM + SC) and HAM x (HAM + SC) groups, with *p*-values of 0.031 and 0.019, respectively.

According to the results in Table 1, significant differences were found when comparing the Control x (HAM + SC) groups for the DV (mL; *p* = 0.031) and SV (mL; *p* = 0.05) variables. In addition, there was also statistical significance when comparing the HAM x (HAM + SC) groups for the DV (mL; *p* = 0.019) variables. However, the result obtained for SV (mL) may have been interfered with by the increase in DV (mL), since the analysis of end-systolic volume is directly related to end-diastolic volume. As a result, the reduction in the mean SV value obtained in the HAM + SC and SC groups on the 30th day of the study may have been influenced by the reduced mean DV value for these groups. This was also observed in the study by Blume et al., which analyzed the use of human amniotic membrane and isolated stem cells after myocardial ischemia, in which there was a reduction in the mean value of both SV and DV in the HAM and SC groups [10].

Histological analysis of the myocardial tissue slides was carried out using Gomori trichrome and Sirius-red stains, which made it possible to measure the extent of myocardial fibrosis and the specification of the collagen present in these areas, respectively. This analysis made it possible to correlate the histological and functional results, since the fibrosis area can be delimited by collagen expression and is associated with deterioration in ventricular function. This is because after myocardial necrosis occurs, fibrosis is established in areas previously occupied by cardiomyocytes [13].

The results obtained from the analysis of the Gomori trichrome variable were statistically significant (*p* = 0.033). It is therefore possible to state that the Control and SC groups showed greater extensions of fibrosis compared to the HAM and HAM + SC groups, according to Table 3. In addition, the analysis of the groups two by two indicated that statistical significance occurred when comparing the Control and HAM + SC groups (*p* = 0.05), as shown in Table 4. As a result, it can be inferred that the use of the biomaterial consisting of amniotic membrane and stem cells led to a reduction in the scar area compared to the Control group, as observed in previous studies [8,11].

As for the analysis of type I and type III collagen (%), the results were statistically significant, with a *p*-value of 0.34 for both. The HAM and HAM + SC groups had lower percentages of type I collagen, compared to the Control and SC groups.

The data obtained from type III collagen already indicates an inverse result, with the HHAM (33.3% ± 25.7) and AM + SC (36.9% ± 32.3) groups showing the highest percentages compared to the Control (9% ± 9.9) and SC (25.4% ± 23.9) groups, as shown in Table 3. In the two-by-two comparison of type I and III collagen variables, statistical significance was found when comparing the Control and AM + SC groups, with a *p*-value of 0.05 for both variables, as shown in Table 4.

Type III collagen is the immature form of type I collagen and has greater elasticity properties [14]. Previous studies have shown that higher levels of type III collagen are related to better cardiac performance in patients with diastolic dysfunction [15]. The difference in the proportion of type I and III collagen in the groups in the areas of infarction is extremely important, as it may indicate different levels of tissue repair. Thus, the higher percentage of type III collagen and lower percentage of type I collagen in the HAM and HAM + SC groups may indicate higher levels of tissue fibrosis reversal than in the Control and SC groups.

Based on the results obtained from the histological analysis, it can be said that the HAM + SC group showed a higher level of tissue repair compared to the Control group. This result indicates greater efficiency of the biomaterial constructed from stem cells and amniotic membrane in reversing tissue fibrosis.

The following markers were used for immunohistochemical analysis: sarcomeric actin, CD31, CD68, desmin and α-actin. α-actin identifies the expression of vascular smooth muscle cells and endothelial cells and is thus involved in the formation of new vessels [16,17]. Similarly, the CD31 marker is related to neoangiogenesis, being highly expressed in endothelial cells, as it constitutes the intercellular junction of these cells [18]. The expression detected by CD68 reflects the infiltration of macrophages into tissues, generally related to the inflammatory response and tissue remodeling processes [19]. Sarcomeric actin and desmin are related to the striated muscle present in the sample. Desmin is considered the main component of the intermediate filament protein expressed in striated and smooth muscle [20].

In this analysis, only α-SMA expression showed statistically significant differences among groups (*p* < 0.001). The HAM and HAM + SC groups exhibited higher percentages of positive staining compared to the Control and SC groups (Table 5). Pairwise comparisons revealed significant differences between Control vs. HAM + SC (*p* = 0.001) and SC vs. HAM + SC (*p* = 0.003) (Table 6). These findings suggest increased activation of α-SMA–positive cells in the HAM + SC group, consistent with enhanced remodeling activity in the infarcted myocardium.

It is important to acknowledge that α-SMA immunostaining alone does not allow definitive discrimination between vascular smooth muscle cells and activated myofibroblasts within the infarcted myocardium. Although morphological features and tissue localization were carefully considered during analysis, the absence of double immunostaining limits precise cellular characterization. Future studies incorporating additional lineage-specific markers would provide a more precise distinction between these cell populations during post-infarction remodeling.

Like the α-actin marker, CD31 is also related to the formation of new blood vessels. In the analysis of the results, the results obtained were not statistically significant (*p* = 0.743), but they did point to a greater predominance of this marker in the HAM + SC group compared to the other Control, SC and HAM groups, as shown in Table 5.

These findings may be related to the potential induction of angiogenesis, myogenesis and neovascularization by the growth factors present in the constructed biomaterial. In turn, these may result in increased tissue repair capacity and therefore improved tissue perfusion, which would be extremely important for the recovery of cardiac function [15,21,22]. In relation to the markers of quantitative expression of striated muscle, desmin and sarcomeric actin, there were similar results between the study groups, with no statistical significance in the data for both variables. In the case of desmin, the HAM and SC groups had the highest percentages of the marker compared to the Control and HAM + SC groups, with a *p*-value of 0.264 (Table 6). In the case of sarcomeric actin, there was greater detection of this marker in the SC and AM groups compared to the HAM + SC and Control groups, with a *p*-value of 0.755.

In addition, this study analyzed macrophage infiltration in myocardial tissue samples through the expression of CD68. In the study by Ryabov et al., a higher percentage of macrophages was detected in the regenerative phase after myocardial ischemia [23]. Thus, in our study, the analysis of CD68 indicated that the mean values obtained for the HAM + SC group were higher than those found for the Control, SC and AM groups, in which there was no statistical significance for the values found (*p* = 0.061), as shown in Table 5.

Pro-inflammatory factors and activated cytokines play crucial roles in the post-myocardial infarction inflammatory response. Previous evidence indicates that macrophages, marked by the expression of CD68, accumulate in injured myocardial tissue, where they actively act in the repair process. This cellular mobilization can significantly influence cardiac remodeling, contributing to the structural and functional recovery of the heart [24,25].

Although our results were not statistically significant, the trend towards greater CD68 expression in the HAM + SC group may suggest a more intense mobilization of macrophages in this group. This response may reflect a more active acute or controlled inflammatory process, compatible with an early-stage regenerative environment. This immune activation may be associated with the combined action of the extracellular matrix of the amniotic membrane and the paracrine effects of the stem cells, potentiating the tissue response to the injury.

In contrast, isolated stem cell therapy may have a reduced impact in the ischemic myocardium, which is characterized by hypoxia, oxidative stress, and inflammatory mediators that create a hostile microenvironment and may impair cell survival and regenerative potential.

The present study is the absence of direct cell-tracking experiments to quantify stem cell retention, survival, or engraftment within the infarcted myocardium. No fluorescent labeling, molecular quantification (e.g., species-specific qPCR), or in vivo imaging techniques were employed to monitor transplanted cells over time. Therefore, the functional and histological improvements observed cannot be definitively attributed to long-term cellular engraftment. Future investigations incorporating cell-labeling strategies and molecular tracking approaches will be essential to better elucidate the fate and persistence of transplanted cells in the post-infarction environment.

Isolated stem cells have less impact due to the expression of environmental factors, since after the occurrence of ischemia, the myocardium is considered a hostile environment for the proper development of stem cells, which reduces the regenerative capacity of these cells [26,27,28]. Thus, the biomaterial constructed from stem cells and amniotic membrane presented superior results to the isolated use of stem cells due to the growth factors present in the amniotic membrane, which provided a suitable environment to effect the regenerative potential of stem cells. These results are supported by Simeoni et al. [29], who also suggested that the effect of a graft as a delivery system could further enhance the effects of the transplanted cells.

This study has some limitations. First, we did not perform direct cell-tracking (e.g., fluorescent or molecular labeling), so the retention, survival, and engraftment of mononuclear cells in the infarcted myocardium remain uncertain, and the balance between sustained engraftment and paracrine effects can only be inferred. Second, left ventricular volumes were measured by two-dimensional B-mode echocardiography using Simpson’s method, which is less precise than cardiac magnetic resonance or high-frequency small-animal ultrasound and may introduce variability in the functional estimates. Future experiments using in vivo cell-tracking and higher-resolution imaging will be important to clarify the fate of transplanted cells and to strengthen functional assessment.

## 4. Materials and Methods

This study was approved by the Animal Use Ethics Committee (CEUA) of the Pontifical Catholic University of Paraná (PUCPR) under protocol number 02285. A total of 120 male Wistar rats (aged 2–3 months) underwent experimental induction of acute myocardial infarction (AMI).

**Figure 7:**
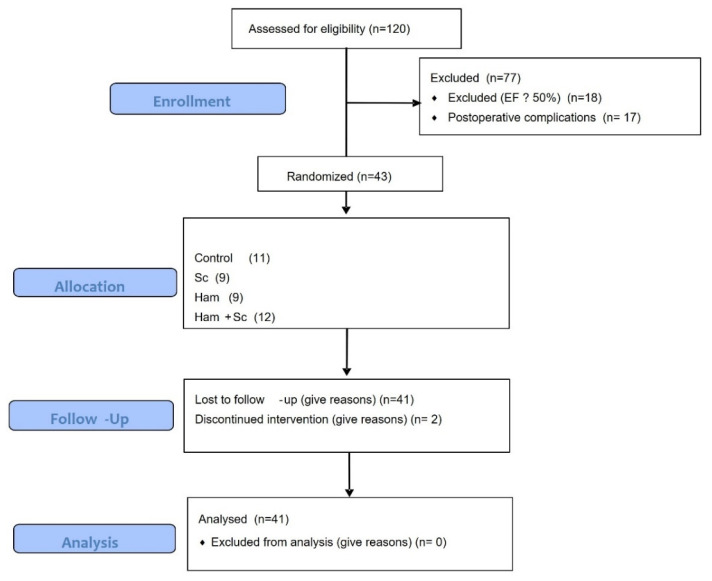
Flowchart of animal inclusion, exclusions, and mortality throughout the experimental protocol.

Following AMI induction, 35 animals died during the perioperative period. On the seventh (7th) day post-infarction, the 85 surviving animals underwent transthoracic echocardiography to assess left ventricular ejection fraction (LVEF). Among these, 43 rats met the inclusion criterion of LVEF < 50% and were considered eligible for the study. These animals were then randomly and evenly assigned to four experimental groups. The overall flow of animal inclusion, exclusions, and mortality throughout the experimental protocol is summarized in Figure 7.

These animals were randomly and evenly assigned to four experimental groups:

- Group I (Control): Injection of sterile saline solution (0.9% NaCl) into the infarcted myocardial region;
- Group II (Stem Cells—SC): Implantation of mononuclear stem cells into the infarcted myocardial region;
- Group III (Amniotic Membrane—HAM): Implantation of human amniotic membrane into the infarcted myocardial region;
- Group IV (Stem Cells + Human Amniotic Membrane—SC + HAM): Combined implantation of mononuclear stem cells and human amniotic membrane into the infarcted myocardial region.

On the 30th day, all surviving animals underwent a second transthoracic echocardiogram and were subsequently euthanized. Myocardial tissue samples were collected for histopathological and immunohistochemical analysis.

### 4.1. Human Amniotic Membrane (HAM) Acquisition

Umbilical cords and placental tissues were obtained from two consenting mothers (gestational age: 36–40 weeks) who signed informed consent forms in accordance with a protocol approved by the Research Ethics Committee of Pequeno Príncipe Hospital (approval numbers 659.204/2014 and 0948-11).

HAM was collected under sterile conditions immediately after placental expulsion in the surgical setting. The collected material included segments of the umbilical cord, chorionic plate, and chorionic villi. All processing procedures, including membrane decellularization, mesenchymal stem cell isolation, culture, and phenotypic characterization, were performed at the Tissue Regeneration Center of PUCPR.

All material handling was carried out under a laminar flow hood to maintain sterility. After removal of residual blood and adherent tissues, the HAM was immersed in phosphate-buffered solution containing chlorine dioxide (ClO_2_), stabilized in an 8% aqueous solution, at a final concentration of 100 ppm, and agitated at room temperature for 20 min. The membrane was subsequently rinsed and stored in phosphate-buffered saline (PBS, pH 7.2).

The processed HAM used in this study consisted exclusively of the amniotic layer, with the chorion carefully removed during preparation. Histological evaluation of representative samples demonstrated an average thickness of approximately 30 µm (range 20-40 µm) after decellularization. The membrane preserved its basement membrane and stromal matrix architecture, providing structural support for application as an epicardial patch.

### 4.2. Decellularization of Umbilical Cords

The decellularization process followed the protocol described by Blume et al. (2021) [10], which involves mechanical agitation and treatment with sodium dodecyl sulfate (SDS) in a BioSAFE Class II biological safety cabinet (Veco^®^) [Campinas, Brazil].

Initially, the membranes were thoroughly rinsed with PBS to remove blood and debris. They were then treated with 0.01% SDS and sodium deoxycholate (SD) for 24 h at 37 °C using a mechanical shaker (Shaking Table 109 M, Nova Ética Ltd., Lemesos, Cyprus). After treatment, the membranes were stored in PBS at 4 °C. To confirm successful decellularization, microscopic analyses under phase contrast and fluorescence using Hoechst staining were conducted, confirming the absence of cellular nuclei.

HAM has naturally low immunogenicity and exhibits anti-inflammatory, antifibrotic, antibacterial, and site-specific angiogenic properties, which support tissue regeneration [21]. The amniotic membrane was processed under sterile conditions, decellularized, washed in trehalose solution, and then frozen at −80 °C. Previous reports indicate that, regardless of preparation and preservation, its general structural properties are largely retained after long-term storage and rehydration. No alterations were observed after rehydration following long-term storage.

The amniotic membrane was processed under sterile conditions, decellularized, and washed in a trehalose solution to protect against freezing damage. It was then frozen under controlled conditions (lyophilized) at –80 °C. Literature reports indicate that regardless of preparation and preservation methods, the general structural properties of the amniotic membrane are largely retained.

Biomaterial implantation was performed 7 days after AMI, following echocardiographic confirmation of left ventricular dysfunction (EF < 50%). This time point was selected to target the early remodeling phase, characterized by active inflammation resolution and initiation of fibrotic deposition, which provides a more stable microenvironment for graft integration and retention [13].

### 4.3. Isolation of Bone Marrow Mononuclear Cells

Bone marrow was collected seven days after AMI via autologous aspiration under general anesthesia. Anesthesia was induced by intramuscular injection of 5% ketamine (Vetanarcol^®^, König do Brasil Ltda, Mairinque, Brazil) at a dose of 50 mg/kg combined with 2% xylazine hydrochloride (Rompun^®^, Bayer S.A., São Paulo, Brazil) at 10 mg/kg.

The animals were placed in lateral decubitus with the upper hind limb flexed and the lower extended. A 5 mL syringe (BD-Plastipak^®^, Paulínia, Brazil) containing 0.2 mL of heparin (5000 IU/mL) was used to aspirate approximately 1 mL of bone marrow from the posterior iliac crest of the femur using a 25 × 821 mm^2^ G1 needle (BD PrecisionGlide^®^, Curitiba, Brazil). The syringes were then labeled for identification.

Mononuclear cells were isolated by density gradient centrifugation (d = 1.077 g/cm^3^) using Ficoll-Hypaque reagent (Sigma, St. Louis, MO, USA).

### 4.4. Characterization and Viability of Stem Cells

Revealed a predominance of CD45+ cells, indicating hematopoietic origin. A CD34+ subpopulation was also observed, consistent with hematopoietic stem or progenitor cells. Additionally, the presence of CD90+ cells suggests the existence of mesenchymal stem cells in the sample. This phenotypic profile is compatible with the expected cellular heterogeneity of the bone marrow mononuclear fraction.

**Figure 8:**
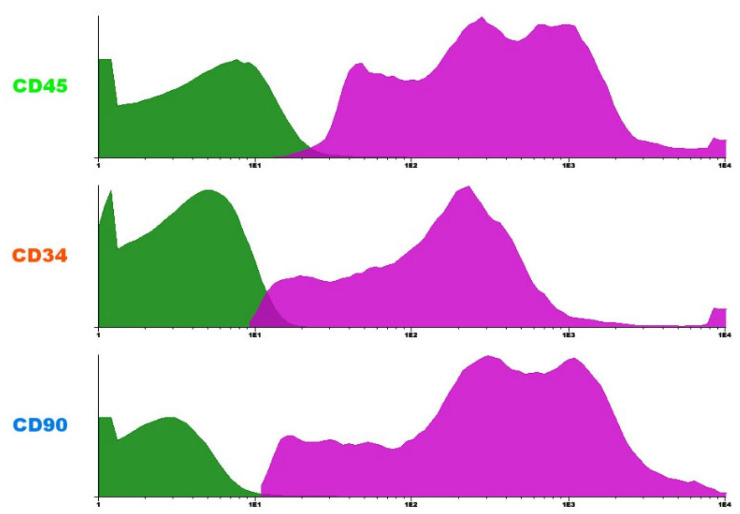
Flow cytometry histograms showing the expression of surface markers CD45, CD34, and CD90 in bone marrow mononuclear cells. CD45+ cells indicate hematopoietic lineage; CD34+ cells represent hematopoietic stem and progenitor cells; and CD90+ cells suggest the presence of mesenchymal stem cells. The magenta peaks represent marker-positive populations, while the green peaks represent marker-negative populations.

Mononuclear cells were characterized by flow cytometry (Figure 8), showing predominant CD45+ cells (75.3 ± 8.9%) and reduced CD14+ expression (12.1 ± 4.2%), consistent with enrichment of hematopoietic progenitors.

### 4.5. Experimental Induction of Acute Myocardial Infarction

**Figure 9:**
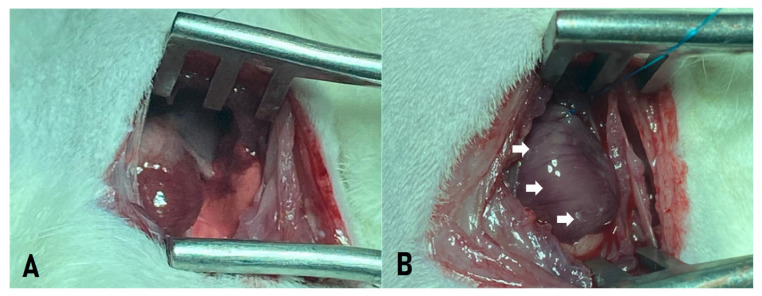
(**A**) Lateral thoracotomy for myocardial access in an animal model; (**B**) myocardial ischemia resulting from ligation of the left coronary artery (white arrow).

The thoracic region was aseptically prepared using povidone-iodine, and a left lateral thoracotomy was performed through the third intercostal space (Figure 9A). After opening the pleura, the animal was connected to a mechanical ventilator (Harvard^®^ model 683, Holliston, MA, USA) using room air (21% O_2_).

The pericardium was opened to allow visualization of the heart. The heart was exteriorized, and the left atrium was mobilized to expose the left coronary artery, which was ligated with a 7-0 non-absorbable polypropylene suture between the pulmonary artery outflow tract and the left atrium. Successful infarction was confirmed by the immediate discoloration of the ischemic area (Figure 9B).

The heart was repositioned, the lungs were hyperinflated, and the chest wall was closed in layers using 4-0 non-absorbable monofilament nylon sutures. After anesthesia recovery, the rats were housed in groups of four per cage and provided with standard chow and water ad libitum.

### 4.6. Biomaterial Transplant

Thus, all implants (SC, HAM, or SC + HAM) were performed 7 days after AMI, following functional confirmation of ventricular dysfunction, and not at earlier time points. This subacute phase corresponds to an active remodeling window, characterized by persistent inflammatory signaling and progressive extracellular matrix reorganization, allowing therapeutic modulation of fibrosis and ventricular geometry.

The procedure was carried out on 43 animals considered eligible for the study, i.e., with LVEF < 50%. Anesthesia was performed with Ketamine 5% (Vetanarcolâ, Konig do Brasil Ltda., São Paulo, Brazil) at a dose of 50 mg/kg associated with Xylazine hydrochloride 2% (Rompunâ, Bayer S.A, São Paulo, Brazil) at a dose of 10 mg/kg, antisepsis of the chest with topical povidone-iodine and median sternotomy, and the animals were reconnected to the mechanical ventilation system. Volume respirators (Harvard^®^, Inc., model 683 respirator, Holliston, MA, USA) were used for small animals, with 21% oxygen (room air). Afterwards, asepsis was carried out with povidone-iodine.

For myocardial implantation, HAM patches were prepared as single-layer amniotic membranes with an approximate thickness of 80–120 µm, as determined in representative histological sections. After decellularization and storage, square grafts measuring 7 × 7 mm^2^ were trimmed from the amniotic sheet, ensuring preservation of the native basement membrane and stromal architecture.

**Figure 10:**
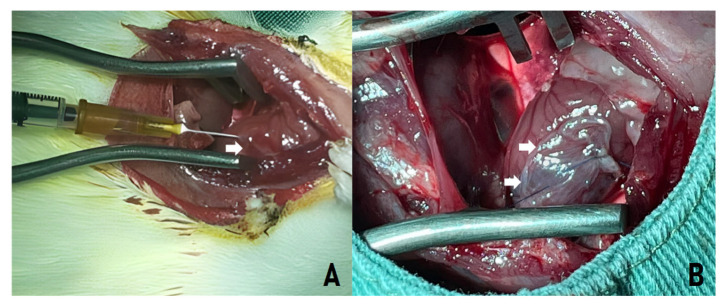
Median Sternotomy. (**A**) Implantation of stem cells (SC) in the myocardium of an animal model (white arrow). (**B**) Human amniotic membrane implant (HAM) in the myocardium of an animal model (white arrow).

- Control Group: three intramyocardial injections were made into the infarcted area with sterile saline solution (0.9% NaCl). After reviewing hemostasis and insufflating the lungs, the chest wall was sutured in planes with 4.0 non-absorbable monofilament mononylon suture.
- SC implantation: Animals in the SC group received approximately 5 × 10^6^ bone marrow mononuclear cells diluted in 100 µL of sterile saline (0.9% NaCl), administered as intramyocardial injections into the peri-infarct region (Figure 10A).
- HAM implantation: a 7 × 7 mm^2^ segment of the HAM was implanted over the ischemic area. The grafts were fixed using prolene 6.0 sutures (Ethicon^®^, Inc., Somerville, NJ, USA) at each end, with the aim of completely covering the infarcted area (Figure 10B). After reviewing hemostasis and insulating the lungs, the chest wall was sutured in planes with 4.0 non-absorbable monofilament mononylon suture.
- SC + HAM implantation: Animals received 5 × 10^6^ bone marrow mononuclear cells diluted in 100 µL of sterile saline (0.9% NaCl) via intramyocardial injections in the peri-infarct region, followed by implantation of a 7 × 7 mm^2^ HAM patch over the ischemic area, fixed with 6-0 prolene sutures to completely cover the infarcted region (Figure 10B).

### 4.7. Echocardiographic Examination

Two-dimensional transthoracic echocardiography was performed using an ultrasound imaging system equipped with S12 (5–12 MHz) and 15L6 (7–15 MHz) sector transducers, specifically designed for small-animal imaging and capable of frame rates up to 160 Hz.

Images were acquired in two-dimensional B-mode using parasternal long-axis and short-axis views. Left ventricular (LV) volumes and ejection fraction were calculated using the modified Simpson’s method from apical-equivalent views adapted for small-animal imaging.

The following parameters were obtained: LV end-diastolic surface area, LV end-systolic surface area, LV end-diastolic length, LV end-systolic length, and heart rate. These measurements were used to calculate end-diastolic volume (DV, mL), end-systolic volume (SV, mL), and left ventricular ejection fraction (LVEF). All measurements were obtained from three consecutive cardiac cycles and averaged for analysis.

**Figure 11:**
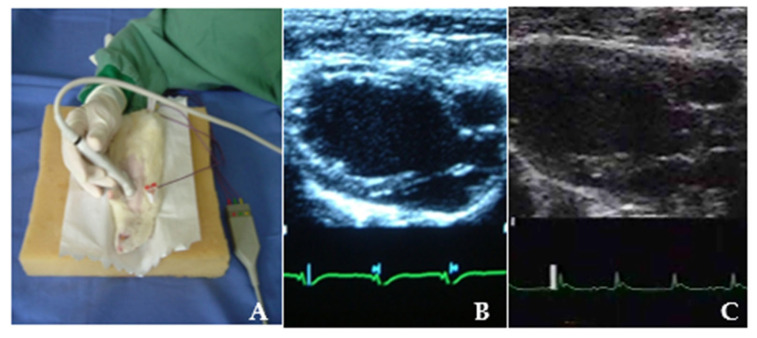
(**A**) Examiner performing an echocardiogram on one of the animals. (**B**) View of the Systolic Volume obtained by echocardiogram from one of the animals. (**C**) View of the Diastolic Volume obtained by echocardiogram from one of the animals.

All analyses were performed by the same experienced cardiologist, blinded to group allocation and study phase. Each parameter was measured three times, and the mean value was used for statistical analysis. This methodology has been widely applied and validated in experimental rat models of myocardial infarction, providing reliable functional assessment under standardized acquisition conditions. Figure 11A illustrates echocardiographic acquisition in one experimental animal, while Figure 11B,C show representative images of end-systolic and end-diastolic volume measurements, respectively.

### 4.8. Histological and Immunohistochemical Analysis

For immunohistochemical analysis, 4 µm paraffin sections were placed on silanized slides, cleared in xylene, and rehydrated through decreasing alcohol concentrations. Antigen retrieval was achieved by heat treatment in either citrate buffer (pH 6.0) for α-actin, desmin, and sarcomeric actin, or EDTA buffer (pH 9.0) for CD31 and CD68, followed by cooling at room temperature. Endogenous peroxidase was quenched with hydrogen peroxide, and nonspecific reactions were reduced using normal serum or bovine serum albumin. The following primary antibodies were used: α-smooth muscle actin (mouse monoclonal, clone 1A4, ab7817, 1:100 Abcam Inc., Toronto, ON, Canada), desmin (mouse monoclonal ab8592; 1:100 Abcam Inc., Toronto, ON, Canada), CD31 (rabbit polyclonal, Abcam, ab9498, 1:50 Abcam Inc., Toronto, ON, Canada), CD68 (mouse monoclonal, ab283654; 1:50, Abcam Inc., Toronto, ON, Canada), and sarcomeric actin (monoclonal ab68168; 1:200, Abcam Inc., Toronto, ON, Canada). Sections were incubated overnight at 4 °C, followed by polymer-based HRP detection, DAB chromogen development, and hematoxylin counterstaining. Appropriate positive control tissues were processed in parallel, and negative controls were generated by omitting the primary antibody or substituting it with isotype-matched immunoglobulin. Slides were evaluated under light microscopy by five blinded observers, and quantitative analysis was performed in ten representative fields per sample using standardized image parameters.

All the animals were euthanized with intravenous sodium pentobarbital at a dose of 200 to 250 mg/kg. With a view to animal welfare, if there were clinical manifestations of the disease such as signs of respiratory depression, abnormal breathing, wheezing and nasal secretion; acute lung edema; factors associated with cardiac pump failure such as decreased peripheral circulation, blue and cold extremities; jaundice; and secretions in the eyes, the animals would be euthanized before the period stipulated by the research.

### 4.9. Statistical Analysis

The results of the EF (%), SV (mL) and DV (mL) variables were described by mean, standard deviation, median, minimum and maximum. The analysis of variance (ANOVA) model with one factor was used to compare the groups in terms of the results of the assessment on day 7 and the differences between the assessments on days 7 and 30 in terms of the variables ejection fraction, systolic volume and diastolic volume. The groups were compared with regard to the results on day 30 using the one-factor analysis of covariance (ANCOVA) model, including the results of the 7-day assessment as a covariate. The Bonferroni post hoc test was used for multiple comparisons after ANOVA and ANCOVA. Student’s *t*-test for paired samples was used to compare the results on day 7 with the results on day 30 within each group. The Kruskal–Wallis non-parametric test was used to compare the groups in terms of the percentage of collagen (type I and type III) and Gomori trichrome. Dunn’s test with Bonferroni adjustment was used for multiple comparisons between groups. The normality of the variables was assessed using the Kolmogorov–Smirnov test. Values of *p* < 0.05 indicated statistical significance. The data was analyzed using the computer program IBM SPSS Statistics v.20.0. Armonk, NY, USA: IBM Corp.

The post hoc analyses (i.e., pairwise comparisons performed after a global test such as ANOVA or Kruskal–Wallis indicates a significant difference) were conducted with multiple comparisons corrections—Bonferroni adjustment following ANOVA and Dunn’s test with Bonferroni correction following Kruskal–Wallis. These corrections multiply the unadjusted *p*-values by the number of pairwise comparisons, which can result in adjusted *p*-values exceeding 1. In such cases, it is standard practice to report *p* = 1, as this is the maximum possible value for a *p*-value.

Paired correlations between changes in functional parameters (ΔEF, ΔSV, and ΔDV) and histological markers of remodeling (Gomori-stained fibrotic area and type I and III collagen percentage) were assessed at the individual animal level. Data normality was evaluated using the Shapiro–Wilk test, and Pearson’s or Spearman’s correlation coefficients were applied as appropriate. Correlation strength was interpreted according to standard criteria, and statistical significance was set at *p* < 0.05.

## 5. Conclusions

It is suggested that the biomaterial composed of mononuclear stem cells and human amniotic membrane was associated with reduced end-diastolic and end-systolic volumes, a smaller fibrotic area, and a shift toward higher type III and lower type I collagen, indicating more favorable ventricular remodeling in this rat model of AMI with left ventricular dysfunction. In addition, increased α-actin expression in the HAM + SC group may reflect enhanced neoangiogenesis and myofibroblast activity in the infarct border zone. These findings support the potential of HAM-based cell delivery as a regenerative strategy, although further studies with longer follow-up and larger cohorts are required.

## Figures and Tables

**Table 1:** Analysis of the FE (%), VS (mL) and VD (mL) variables between the study groups.

Variable	Group (*n*)	Average ± SD	*p* * (Comparison of the 4 Groups)
**EF (%) Day 7**	CONTROL (12)	37.3 ± 5.6	0.349
	HAM (10)	35.1 ± 5.9	
	SC (9)	38.9 ± 6.5	
	HAM + SC (12)	37.9 ± 6	
**EF (%) Day 30**	CONTROL (12)	36.5 ± 5.7	0.072
	HAM (10)	37.8 ± 9.3	
	SC (9)	33.3 ± 7.1	
	HAM + SC (12)	42.2 ± 9	
**SV (mL) Day 7**	CONTROL (12)	0.159 ± 0.046	0.349
	HAM (10)	0.143 ± 0.067	
	SC (9)	0.191 ± 0.066	
	HAM + SC (12)	0.177 ± 0.067	
**SV (mL) Day 30**	CONTROL (12)	0.194 ± 0.07	0.023
	HAM (10)	0.194 ± 0.071	
	SC (9)	0.156 ± 0.075	
	HAM + SC (12)	0.118 ± 0.068	
**DV (mL) Day 7**	CONTROL (12)	0.257 ± 0.065	0.533
	HAM (10)	0.257 ± 0.142	
	SC (9)	0.315 ± 0.098	
	HAM + SC (12)	0.287 ± 0.096	
**DV (mL) Day 30**	CONTROL (12)	0.301 ± 0.079	**0.005**
	HAM (10)	0.313 ± 0.077	
	SC (9)	0.229 ± 0.105	
	HAM + SC (12)	0.197 ± 0.084	

* ANOVA with one factor (D7 and Differ); ANCOVA with one factor including D7 as a covariate; *p* < 0.05. Legend: EF: Ejection Fraction; SV: Systolic Volume; DV: Diastolic Volume; SD: Standard Deviation; HAM: Human Amniotic Membrane; SC: Stem Cells.

**Table 2:** Analysis of the two by two groups for the variables SV (mL) And DV (mL).

*p* *		Compared Groups
VS (mL) D30	VD (mL) D30	
1	1	CONTROL X HAM
1	0.378	CONTROL X SC
0.050	0.031	CONTROL X (HAM + SC)
1	0.235	HAM x SC
0.093	0.019	HAM x (HAM + SC)
1	1	SC x (HAM + SC)

* Student’s *t*-test for paired samples, *p* < 0.05. Adjusted *p*-values using Bonferroni correction. Values greater than 1 were reported as 1. Legend: HAM: Human Amniotic Membrane; SC: Stem Cells.

**Table 3:** Analysis of the variables Gomori Trichrome (µM^2^), Collagen Type I (%) And TYPE III (%) Between the Study Groups.

*p* *	Average ± SD	Group (*n*)	Variable
(Comparison of the 4 Groups)			
	15,585,072 ± 7,997,958	CONTROL (11)	GOMORI TRICHROME (µm^2^)
	12,810,354 ± 2,452,308	SC (9)	
	8,890,165 ± 5,613,027	HAM (9)	
**0.033**	8,961,009 ± 5,283,955	HAM + SC (12)	
	91.0 ± 9.9	CONTROL (11)	TYPE I COLLAGEN (%)
	74.6 ± 23.9	SC (9)	
	66.7 ± 25.7	HAM (9)	
**0.034**	63.1 ± 32.3	HAM + SC (12)	
	9 ± 9.9	CONTROL (11)	TYPE III COLLAGEN (%)
	25.4 ± 23.9	SC (9)	
	33.3 ± 25.7	HAM (9)	
**0.034**	36.9 ± 32.3	HAM + SC (12)	

* ANOVA with one factor or Kruskal–Wallis non-parametric test; *p* < 0.05. Legend: SD: Standard Deviation. Legend: SD: Standard Deviation; HAM: Human Amniotic Membrane; SC: Stem Cells.

**Table 4:** Analysis of the two by two groups for the variables gomori tricronic (µM^2^), Collagen Type I (%) and Type III (%).

*p* *			Compared Groups
Type III	Type I	Gomori	
Collagen	Collagen		
0.428	0.428	1	CONTROL X SC
0.083	0.083	0.093	CONTROL X HAM
**0.050**	**0.050**	**0.050**	CONTROL X (HAM + SC)
1	1	0.933	SC X HAM
1	1	0.819	SC X (HAM + SC)
1	1	1	HAM X (HAM + SC)

* Bonferroni post hoc test or Dunn test, *p* < 0.05. Adjusted *p*-values using Bonferroni correction. Legend: HAM: Human Amniotic Membrane; SC: Stem Cells.

**Table 5:** Analysis of the variables α-ACTIN (%), CD31 (%), CD68 (%), DESMIN (%) and SARCOMERIC ACTIN (%) between the study groups.

Variable	Group	Average ± SD	*p* * (Comparison of the 4 Groups)
α-ACTIN (%)	CONTROL (11)	1.97 ± 1.61	
	SC (9)	2.38 ± 2.20	
	HAM (9)	3.49 ± 2.19	
	HAM + SC (12)	13.32 ± 10.21	**<0.001**
CD31 (%)	CONTROL (11)	0.44 ± 0.43	
	SC (9)	0.24 ± 0.23	
	HAM (9)	0.32 ± 0.25	
	HAM + SC (12)	0.55 ± 0.67	0.743
CD68 (%)	CONTROL (11)	0.37 ± 0.40	
	SC (9)	0.94 ± 1.30	
	HAM (9)	0.36 ± 0.66	
	HAM + SC (12)	1.09 ± 0.89	0.061
DESMIN (%)	CONTROL (11)	0.80 ± 0.71	
	SC (9)	1.77 ± 2.83	
	HAM (9)	2.37 ± 3.71	
	HAM + SC (12)	0.91 ± 1.51	0.264
SARCOMERIC ACTIN (%)	CONTROL (11)	2.58 ± 2.02	
	SC (9)	4.02 ± 5.43	
	HAM (9)	3.55 ± 2.30	
	HAM + SC (12)	3.28 ± 3.15	0.755

* Nonparametric Kruskal–Wallis test; *p* < 0.05. Legend: SD: Standard Deviation; HAM: Human Amniotic Membrane; SC: Stem Cells.

**Table 6:** Analysis of the two by two groups for the variable **α**-ACTIN (%).

*p* *	Compared Groups
α-ACTIN (%)	
1	CONTROL X SC
1	CONTROL X HAM
**0.001**	CONTROL X (HAM+SC)
1	SC X HAM
**0.003**	SC X (HAM + SC)
0.066	HAM X (HAM + SC)

* Bonferroni post-hoc test or Dunn test, *p* < 0.05. Adjusted *p*-values using Bonferroni correction. Legend: HAM: Human Amniotic Membrane; SC: Stem Cells.

## Data Availability

The original contributions presented in this study are included in the article. Further inquiries can be directed to the corresponding author.
